# Strongly correlated oxides for energy harvesting

**DOI:** 10.1080/14686996.2018.1529524

**Published:** 2018-11-26

**Authors:** Jobu Matsuno, Jun Fujioka, Tetsuji Okuda, Kazunori Ueno, Takashi Mizokawa, Takuro Katsufuji

**Affiliations:** a Department of Physics, Osaka University, Osaka, Japan; b Center for Emergent Matter Science (CEMS), RIKEN, Saitama, Japan; c PRESTO, Japan Science and Technology Agency, Kawaguchi, Saitama, Japan; d Department of Applied Physics, University of Tokyo, Tokyo, Japan; e Graduate School of Pure and Applied Science, University of Tsukuba, Tsukuba, Japan; f Graduate School of Science and Engineering, Kagoshima University, Kagoshima, Japan; g Department of Basic Science, University of Tokyo, Tokyo, Japan; h Department of Applied Physics, Waseda University, Tokyo, Japan; i Department of Physics, Waseda University, Tokyo, Japan; j Kagami Memorial Laboratory for Material Science and Technology, Waseda University, Tokyo, Japan

**Keywords:** Thermoelectric materials, strongly correlated oxides, Seebeck effect, spin Hall effect, spin Seebeck effect, anomalous Nernst effect, Weyl semimetal, Dirac semimetal, magnetic skyrmion, 50 Energy Materials, 210 Thermoelectronics / Thermal transport / insulators

## Abstract

We review recent advances in strongly correlated oxides as thermoelectric materials in pursuit of energy harvesting. We discuss two topics: one is the enhancement of the ordinary thermoelectric properties by controlling orbital degrees of freedom and orbital fluctuation not only in bulk but also at the interface of correlated oxides. The other topic is the use of new phenomena driven by spin-orbit coupling (SOC) of materials. In 5*d* electron oxides, we show some SOC-related transport phenomena, which potentially contribute to energy harvesting. We outline the current status and a future perspective of oxides as thermoelectric materials.

## Graphical Abstract

**Figure:**
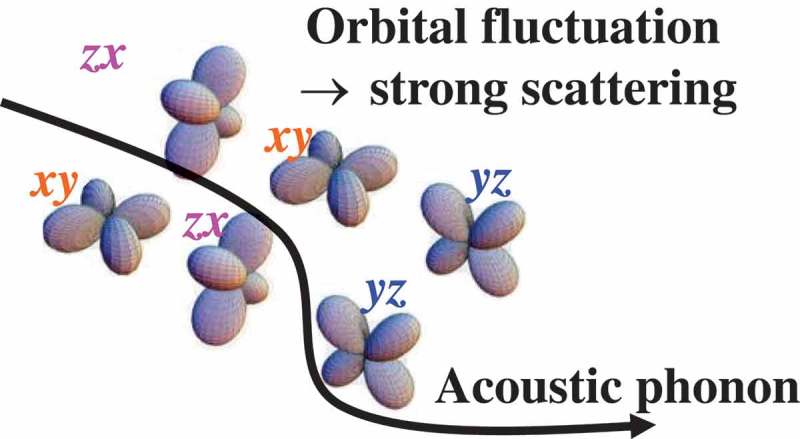

## 1. Introduction

Strongly correlated oxides have been studied extensively for almost three decades since the discovery of high-$T_{c}$ cuprates. A rich variety of physical properties and/or functionalities have been found, for example, colossal magnetoresistance, multiferroics, resistive random access memory. Such versatility of strongly correlated oxides leads to the expectation that they can provide new approaches for energy harvesting. Indeed, several transition-metal oxides were proposed as promising thermoelectric materials [1,2]; the degeneracy in the *d* orbital of the transition-metal contributes to the entropy of the system, resulting in the large Seebeck coefficient.

In this article, we review recent advances in oxides as energy harvesting materials. Our goal is to efficiently convert temperature differences into electric power. Here we discuss two approaches that use transition-metal oxides. One aims to enhance the ordinary thermoelectric properties by tuning the physical properties of correlated oxides. In order to enhance the dimensionless figure of merit for thermoelectric materials *ZT*, one has to increase the Seebeck coefficient and/or to reduce phonon thermal conductivity. We discuss how to control orbital degrees of freedom and orbital fluctuation not only in bulk but also at the interface of correlated oxides for these purposes.

The other approach is to use new phenomena such as spin Seebeck effect and anomalous Nernst effect for energy conversion. In both phenomena, a voltage appears perpendicular to the temperature gradient, in striking contrast to ordinary Seebeck effect, where the voltage appears always along the direction of the temperature gradient. Accordingly, the voltage and the power generated by these phenomena scale with the device area, and this is a great advantage in large-area applications. Since both phenomena are dominated by spin-orbit coupling (SOC) of materials, oxides with heavy transition metal will produce a larger effect. We also discuss other SOC-related phenomena, which potentially contribute to energy harvesting.

We aim to show the current status of correlated oxides as thermoelectric materials. In most of the materials mentioned in this article, the performance does not reach a ready-to-use level; even the underlying mechanisms are not fully clarified. We expect that understanding those mechanisms will lead to a substantial enhancement of the performance as thermoelectric materials and that those materials will be used for energy harvesting in near future.

## 2. Thermoelectric properties in strongly correlated oxides

### 2.1. Orbital degeneracy and seebeck coefficient

The dimensionless figure of merit for thermoelectric materials ZT, where *T* is the temperature of the material, is given by the Seebeck coefficient *S*, the electrical conductivity σ, and the thermal conductivity κ of the material, as $ZT=S^{2}\sigma T/\kappa$. This means that a larger Seebeck coefficient, a larger electrical conductivity, and a smaller thermal conductivity lead to a larger figure of merit. Here, thermal conductivity is given by the sum of the electronic thermal conductivity and the phonon thermal conductivity of the material. Since the electronic thermal conductivity is proportional to the electrical conductivity with a proportionality constant of *LT*, where $L=(\pi^{3}/3)(k_{B}/e{)}^{2}$ (the Lorenz number) is a universal constant, the electronic thermal conductivity cannot be reduced without reducing the electrical conductivity. Thus, it is important to reduce the phonon thermal conductivity of the material in an attempt to enhance the figure of merit ZT.

It is known that degeneracy of the *d* orbitals leads to a large Seebeck coefficient of the material [2]. This was originally proposed to explain the large positive Seebeck coefficient of NaCo_2_O_4_ [1], where a small number of holes exist in the fully occupied triply degenerate $t_{2}{\;}_{g}$ state of the Co ion. It was shown that in the limit of the localized picture, the Seebeck coefficient is proportional to the number of states for all the sites, and if there are energetically degenerate orbitals at each site, the number of states is multiplied by the number of degeneracy and as a result, the system can have a larger Seebeck coefficient. More quantitatively, the Seebeck coefficient is given by the so-called extended Heikes formula;

(1) $$S=\frac{k_{B}}{e}\log\frac{g_{A}}{g_{B}}\frac{x}{1-x},$$

where $k_{B}$ is the Boltzmann constant, 0<x<1 is the number of carriers per site, and $g_{A}$ and $g_{B}$ are the numbers of states per site with and without carrier, respectively. It should be pointed out that in normal metals, this formula does not hold because the number of states is substantially reduced by the Fermi degeneracy. In this sense, strong correlation between *d* electrons is essential to the large Seebeck coefficient in NaCo_2_O_4_. It was also proposed theoretically that the ‘pudding mold’ type band structure contributes to the large Seebeck coefficient in NaCo_2_O_4_ [3], and such a band structure was experimentally observed [4].

In addition to NaCo_2_O_4_, various cobalt oxides were investigated and found to exhibit large positive Seebeck coefficient, and thus can be good p-type thermoelectric materials [5–7]. On the other hand, the n-type version of this kind of materials has not been very much studied. One example of such materials is doped SrTiO_3_ with a perovskite structure [8,9], where a small number of electrons exist in the $t_{2g}$ state of the Ti ion. By varying the concentration of electrons n with La doping to the Sr site, it was found that the figure of merit ZT at room temperature reaches 0.1 [8]. The maximum ZT appears at very low carrier concentration (n∼0.02), where electron correlation is supposedly not very strong. It has also been reported that the Seebeck coefficient is enhanced in the two-dimensional structure [9] with the highly itinerant character of electrons in SrTiO_3_.

**Figure 1.**
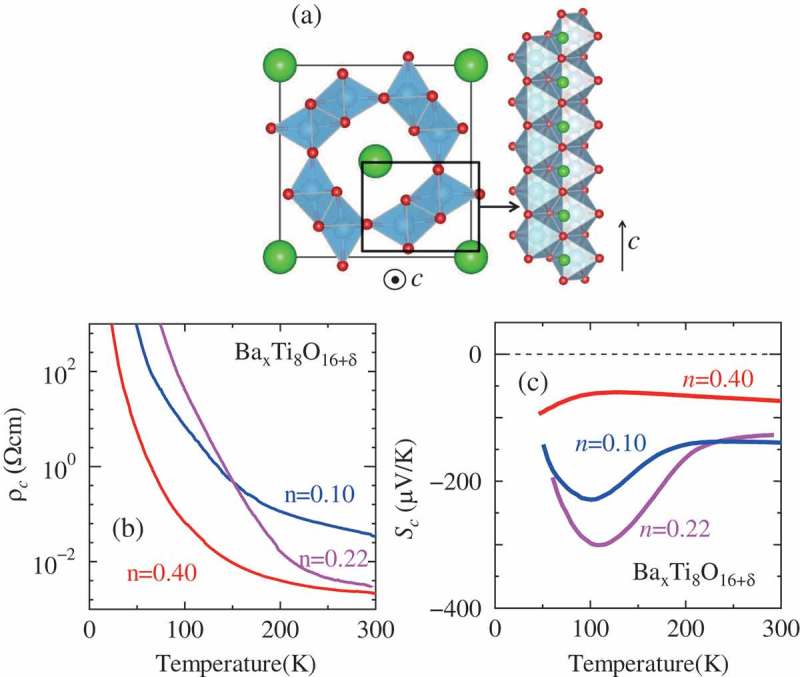
(a) Crystal structure of hollandite $Ba_{x}Ti_{8}O_{16+\delta }$. (b)(c) Temperature dependence of (b) the resistivity and (c) the Seebeck coefficient along the *c* axis (the chain direction) for $Ba_{x}Ti_{8}O_{16+\delta }$ with various values of *n*. Reproduced with permission from [10,11].

Hollandite titanate, $Ba{\;}_{x}Ti_{8}O_{16+\delta }$ is another example where thermoelectric properties have been investigated with the variation of carrier concentration *n* [10,11]. This series of compounds has one-dimensional Ti-O chains (Figure 1(a)) and exhibits one-dimensional electrical conduction along the chain. The electrical resistivity along the chain direction at 300 K is less than 10^–2^Ωcm and the absolute value of the Seebeck coefficient is larger than 100 *μ*V/K. By varying carrier concentration *n*, which is determined by the number of Ba *x* and the amount of oxygen non-stoichiometry *δ*, the *T* dependence of the resistivity and the Seebeck coefficient changes as shown in Figure 1(b). Together with the result of the thermal conductivity as discussed below, the figure of merit *ZT* at 300 K is maximized at *n* = 0.22 (*ZT*~0.05). According to photoemission spectroscopy, the low-energy electronic structure of this series of compounds is dominated by the polaron state, and the experimental absolute values of the Seebeck coefficient are consistent with those calculated with the Mott formula for the Seebeck coefficient, where the density of states is obtained from a photoemission spectrum [12].

### 2.2. Orbital fluctuation and thermal conductivity

Another way to enhance the figure of merit for thermoelectric materials is to reduce the phonon (lattice) thermal conductivity. The most common strategy to reduce the phonon thermal conductivity is to use heavy elements, since the phonon velocity becomes lower with heavier mass of atoms [13]. The second strategy is to introduce a nanostructure to the material that can reduce the propagation of phonons [14,15]. The third one is to utilize underconstrained crystal structures in which a peculiar mode (the so-called ‘rattling’ mode) can disturb phonon propagation [16,17]. The fourth one is to use the fluctuation of the orbital or charge degree of freedom, which will be discussed in this section.

Let us first discuss the orbital and charge degrees of freedom itself before discussing their fluctuation. As discussed above, there is degeneracy of states for the *d* orbitals in transition metals, for example, three-fold degeneracy in the $t_{2g}$ states and the two-fold degeneracy in the $e_{g}$ states. If there is an electron in such degenerate states, the electron can choose which states to occupy because energetically they are equivalent, and this is called orbital degree of freedom. The interaction between the orbital degrees of freedom at the neighboring sites arises from the second order perturbation energy given by the transfer integral of electrons *t*, the on-site Coulomb repulsion *U*, and the Hund coupling $J_{H}$ (Kugel-Khomskii interaction [18]).With this interaction, the orbital degree of freedom orders at low temperatures (orbital ordering). Furthermore, if the average valence of the transition metal is not integer, each transition metal can take one of the two (or more) different valences, and this is called charge degree of freedom. This can also order at low temperatures (charge ordering), where the *d* electrons of the transition metals are periodically aligned [19,20]. In some compounds, the orbital ordering and the charge ordering occur simultaneously [21].

**Figure 2.**
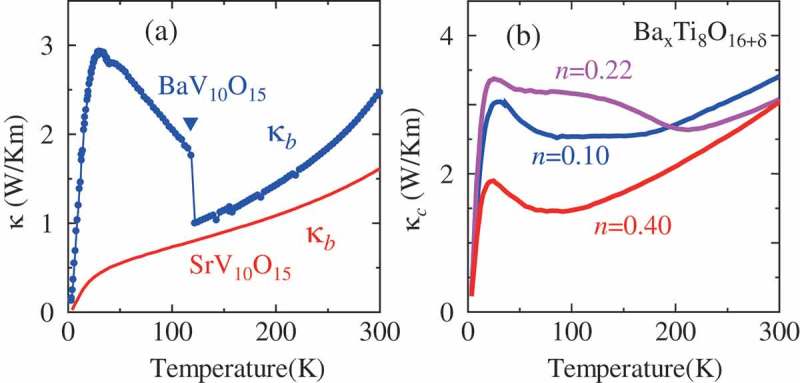
(a)(b) Temperature dependence of the thermal conductivity for (a) BaV_10_O_15_ and SrV_10_O_15_ and (b) $BaTi_{8}O_{16+\delta }$. Reproduced with permission from [11,29].

If there is an orbital/charge ordering, there should be the fluctuation of the orbital/charge degrees of freedom above the transition temperature. It was found that such orbital/charge fluctuation and their ordering affect the phonon thermal conductivity of materials. Namely, above the transition temperature of the orbital/charge ordering, the thermal conductivity is suppressed because the phonons that carry heat are scattered by the fluctuation of the orbital or charge, whereas once the orbital/charge ordering occurs, the source of the phonon scattering disappears and the thermal conductivity is enhanced accordingly [22–29]. Figure 2(a) shows one example of such behavior. BaV_10_O_15_ exhibits an orbital ordering of the $t_{2g}$ states at ∼130 K accompanied by a trimerization of the V ions [30]. On the other hand, SrV_10_O_15_ does not exhibit any ordering or a phase transition down to the lowest temperature [31]. The thermal conductivity for BaV_10_O_15_ exhibits a large jump and further enhancement below the transition temperature (∼130 K) associated with the orbital ordering [29], but such an anomaly is not observed and the thermal conductivity keeps decreasing down to the lowest temperature for SrV_10_O_15_. As a result, the thermal conductivity of SrV_10_O_15_ becomes only ∼15% that of BaV_10_O_15_ at low temperatures.

This suppression of the phonon thermal conductivity can be utilized to enhance the figure of merit for the thermoelectric material, $ZT=S^{2}\sigma T/\kappa$. For example, as shown in Figure 2(b), the phonon thermal conductivity of the hollandite $Ba{\;}_{x}Ti_{8}O_{16+\delta }$ with n=0.22 decreases with decreasing temperature, but starts increasing below ∼200 K [10]. In this compound, a charge ordering of the *d* electrons at Ti sites occurs below ∼200 K, where *d* electrons are located at every five Ti ions along the chain [10], and this charge ordering is responsible for the anomalies of various quantities at ∼200 K shown in Figure 1. Accordingly, the phonon thermal conductivity above 200 K is suppressed by the fluctuation of the charge ordering, but it is enhanced with the charge ordering below 200 K. Compared with doped SrTiO_3_, whose thermal conductivity at room temperature is n∼0.02 W/Km and further increases with decreasing temperature, the phonon thermal conductivity of the hollandite titanates is one third smaller. This leads to a large figure of merit for thermoelectric materials in the hollandite $Ba_{x}Ti_{8}O_{16+\delta }$.

### 2.3. Orbital degree of freedom and interfaces

Another way of utilizing the orbital degree of freedom to reduce the phonon thermal conductivity is to build an interface. From this perspective, the phonon thermal conductivity of the multilayer thin films of SrTiO_3_-SrVO_3_ was investigated [32]. For this series of multilayer thin films with interfaces, the two compounds on both sides of the interface have the same crystal structure (perovskite structure) and a similar lattice constant. Furthermore, since V is placed next to Ti in the periodic table, they have a similar mass density, and the phonon dispersion relation are quite similar (thus, the velocities of the phonons are similar) between the two compounds. In such a case, the phonons propagating in the compounds are barely reflected at the interface, and thus, there should be no additional thermal resistance at the interface. This is equivalent to the light propagating in two materials with an interface; if the two materials have the same refractive index (and thus, the speed of the light does not change across the interface), the light is not reflected at the interface.

**Figure 3.**
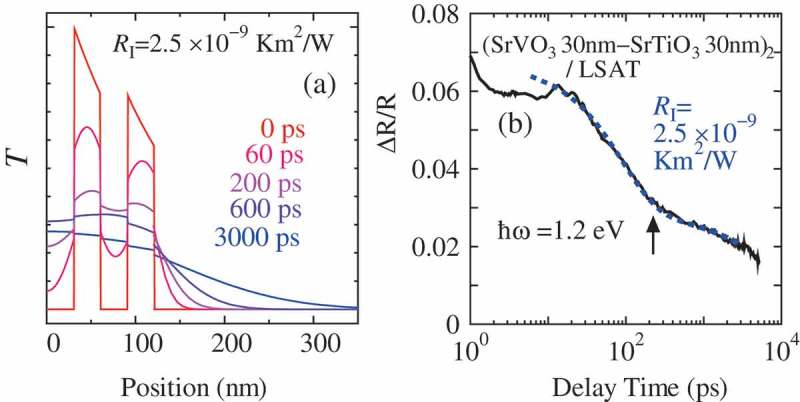
(a) Calculated variation in the temperature after a pump pulse is applied to (SrVO_3_ 30 nm–SrTiO_3_ 30 nm)_2_ on (La,Sr)(Al,Ta)O_3_ (LSAT) substrate, where the interface thermal resistance between SrVO_3_ and SrTiO_3_$R_{I}=2.5\times 10^{-9}$ Km^2^/W is assumed. (b) Experimental time dependence of the reflectivity change at ℏω=1.2 eV for (SrVO_3_ 30 nm–SrTiO_3_ 30 nm)_2_ (a solid line) and the calculated result based on (a) (a dashed line). Reproduced with permission from [32].

In order to measure the thermal conductivity of thin films on the substrate, a conventional method, where the thermal current is applied by a heater attached to the sample and the difference in the temperature along the direction of the thermal current is measured, does not work, since most of the thermal current flows inside the substrate. To overcome this problem, thermoreflectance technique is often used [33–37]. In this technique, the first laser pulse is applied to increase the temperature of the thin film and then the second laser pulse is applied and the intensity of the reflected light is measured as a function of the delay time between the first and the second pulse, assuming that the optical constant of the material changes proportionally to the increase in the temperature. In the conventional thermoreflectance technique, a metal film is deposited onto the thin film that acts as the absorber of the light, but in the case of the SrTiO_3_-SrVO_3_ multilayer, since SrTiO_3_ is transparent but SrVO_3_ is opaque in the visible-light range, the light is absorbed only by the SrVO_3_ layers. This results in the comb-shape temperature profile immediately after the first pulse is applied, and leads to a peculiar time evolution of the temperature in the multilayer (Figure 3(a)) and of the reflectivity change accordingly.

Figure 3(b) shows the reflectivity change as a function of time in a log scale. A kink is observed in the data (shown by an arrow), which corresponds to the time when the comb-shape temperature profile disappears with thermal conduction (see Figure 3(a)). This peculiar time dependence makes it possible to estimate the precise thermal conductivity of the material. Using this novel technique, it was found that the thermal resistance of $∼2\times 10^{-9}$ Km^2^/W exists at the interface between SrTiO_3_ and SrVO_3_, irrespective of the thickness of the layers [32]. This is comparable to or even larger than the thermal resistance between the materials with different phononic properties [38,39].

It is known that at the interface of the transition-metal oxides, the reconstruction of the *d* orbital occurs and the electronic structure can be different from that of the bulk material [40]. In the discussed SrTiO_3_-SrVO_3_ multilayer, where there is no *d* electron in Ti and one *d* electron in the $t_{2g}$ states of V, a phase transition from a metallic state to a Mott-insulating state with decreasing the thickness of SrVO_3_ was observed [41,42]. One possible scenario for the large interface thermal resistance between SrTiO_3_ and SrVO_3_ is that such a reconstruction of the *d* orbital at the interface causes not only the change in the electronic structure but also the change in the phononic properties, resulting in the substantial scattering of phonons at the interface.

## 3. Novel phenomena in heavy transition-metal oxides towards energy harvesting

### 3.1. Correlated Weyl/Dirac semimetal

Topological material has been a subject of intensive research in the modern solid state physics. In topological semimetals (Dirac semimetal or Weyl semimetal), highly mobile relativistic (Dirac/Weyl) electrons with massless or small mass character show up in bulk and offer a playground to study novel electrical and thermal functions. For example, high-mobility carrier is one of preferential features to fabricate excellent thermoelectric device as already demonstrated in conventional materials. Moreover, the quantal phase (Berry phase) often causes unusual electromagnetic response such as giant anomalous Hall/Nernst-effect via the ‘fictitious magnetic field’ in momentum space. Recent research successively clarified various candidate materials including the chalcogenides, pnictides, organic salts and transition-metal oxides. Among them, the 5*d* transition-metal oxide provides an opportunity to study the fundamental physics of magnetism or strong correlation effect of Dirac/Weyl electron. Moreover, the fabrication technique of atomically controlled interface or thin film of oxides has been established in these decades, which provides various possibilities to develop thermoelectric or spin devices. Here, we briefly review the recent research on the correlated Dirac/Weyl semimetal of iridium oxide and present perspective for thermoelectric functions.

**Figure 4.**
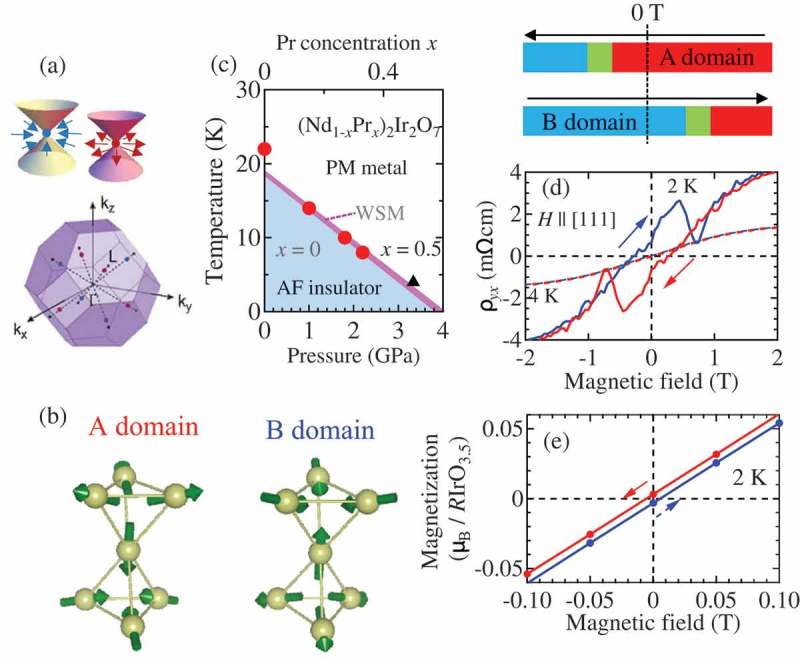
(a) Schematic view of Weyl band dispersion and structure of Weyl points in momentum space for (Nd_0.5_Pr_0.5_)_2_ Ir_2_O_7_. Red and blue symbols denote the Weyl points. (b) Two variants of all-in all-out magnetic structure (c) Electronic phase diagram for Nd_2_Ir_2_O_7_ under the hydrostatic pressure and (Nd_0.5_Pr_0.5_)_2_Ir_2_O_7_. (d) Hall resistivity and (e) magnetization for (Nd_0.5_Pr_0.5_)_2_Ir_2_O_7._

As schematically shown in Figure 4(a), Weyl semimetal (WSM) possesses gapless band dispersion without spin degeneracy in bulk and can emerge in materials without time reversal symmetry (TRS) or spatial inversion symmetry (SIS). The band crossing point (Weyl node) corresponds to the magnetic monopole or anti-monopole in momentum space, which acts as the source or sink of fictitious magnetic field. The pyrochlore-type $R_{2}Ir_{2}O_{7}$ (*R* = rare-earth ion) is a candidate of magnetic Weyl semimetal. Specifically, recent theories propose that the antiferromagnetic Weyl semimetallic state shows up on the verge of metal-insulator transition (Mott transition) [43,44]. For example, Nd_2_Ir_2_O_7_ (single crystal) shows a thermally driven metal-insulator transition (MIT) at 30 K, simultaneously with the antiferromagnetic ordering with ‘all-in all-out’ structure (Figure 4(b)). Recent research clarified that the magnetic transition temperature ($T_{N}$) can be tuned by changing *R*-ion radius or application of pressure and the magnetic Weyl semimetallic phase emerges in a narrow temperature range below $T_{N}$ as shown in Figure 4(c) [45]. Indeed, sizable anomalous Hall resistivity originating from Weyl nodes is observed nearby $T_{N}$ (Figure 4(d)) [46]. We note that the anomalous Hall resistivity is comparable with that in typical metallic ferromagnet [47], while the spontaneous magnetization due to tiny tilting of spin is as small as about 3 m$\mu_{B}$/*R*IrO_3.5_ (Figure 4(e)). Such an enhanced anomalous Hall response is characteristic of giant fictitious magnetic field inherent to magnetic Weyl semimetal. Indeed, recent studies also demonstrate a sizable anomalous Hall effect (AHE) in antiferromagnetic metals with small spontaneous magnetization [48]. Conventionally, it has been believed that the AHE or anomalous Nernst effect is specific in ferromagnetic metals with sizable spontaneous magnetization. In this context, the sizable AHE in antiferromagnets is beyond the conventional wisdom, demonstrating that the concept of Weyl electron may be a useful guideline to develop new materials with large anomalous Hall or anomalous Nernst effect.

**Figure 5.**
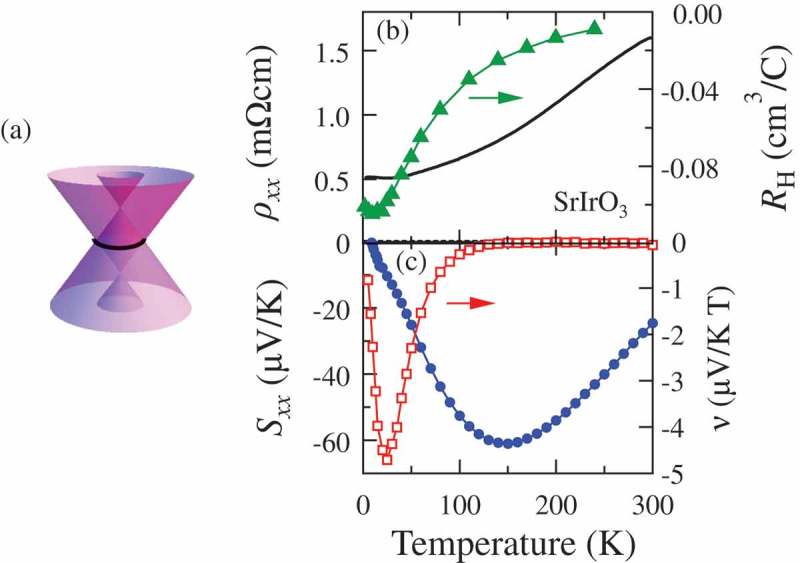
(a) Schematic of Dirac band dispersion for SrIrO_3_. Temperature dependence of (b) resistivity and Hall coefficient (replotted from [51]) and (c) Seebeck and Nernst coefficient for polycrystalline SrIrO_3._

Dirac semimetal is characterized by the gapless band dispersion with spin degeneracy in bulk. In contrast with the Weyl semimetal, the TRS and SIS are required for the Dirac semimetal. Here, we focus on the perovskite *A*IrO_3_ (A = Sr, Ca), which is a candidate of correlated Dirac semimetal. Recent theoretical studies propose the Dirac dispersion with nodal line, wherein the two bands cross in a closed loop in a momentum space as schematically shown in Figure 5(a) [49]. Indeed, the *k*-linear band dispersion is identified by angle resolved photoemission spectroscopy in SrIrO_3_ [50]. As shown in Figure 5(b), the polycrystalline SrIrO_3_ shows a metallic behavior and the Hall coefficient also shows sizable temperature dependence [51]. Figure 5(c) shows the temperature dependence of Seebeck coefficient and Nernst coefficient. The absolute value of Seebeck coefficient $\left|S_{xx}\right|$ reaches 60 μV/K at 150 K. Moreover, the absolute value of Nernst coefficient ν exceeds 4 μV/KT at 25 K. These values are comparable with those for the typical Dirac semimetal Cd_3_As_2_ [52,53], suggesting that the semimetallic state with small carrier density is realized in this material. Although striking feature of thermoelectric response inherent to Dirac electron remains to be elusive, the realization of semimetallic state in perovskite oxide, which is one of standard materials of oxide electronics, may be promising for the future development of oxide thermoelectric device.

### 3.2. Inverse spin hall effect of IR oxides

SOC is a relativistic effect and relates the spin moment of an electron to its orbital momentum via a momentum-dependent effective magnetic field. In the presence of SOC, a charge current without any spin polarization can be converted into a pure spin current (the flow of spin angular momentum) and vice versa, known as the direct spin Hall effect and the inverse spin Hall effect (ISHE) [54–57]. Spin current-based electronics with low-energy consumption has since been discussed, where spin-current injection and detection, using charge/spin conversion by direct spin Hall effect and ISHE, played a key role. Spin Seebeck thermoelectric device is one of the promising applications; the spin current generated in a magnetic layer by temperature gradient is injected into an attached nonmagnetic layer, where the spin current is converted into the voltage with the help of ISHE. Materials with higher charge/spin conversion efficiency are required there. A variety of materials have subsequently been explored and heavy transition metals such as Pt [58] and Au [59] were found to exhibit a particularly large spin Hall angle $\theta_{SH}$ (the maximum yield of the charge/spin conversion), 0.01–0.1 at room temperature, owing to their pronounced SOC effects.

**Figure 6.**
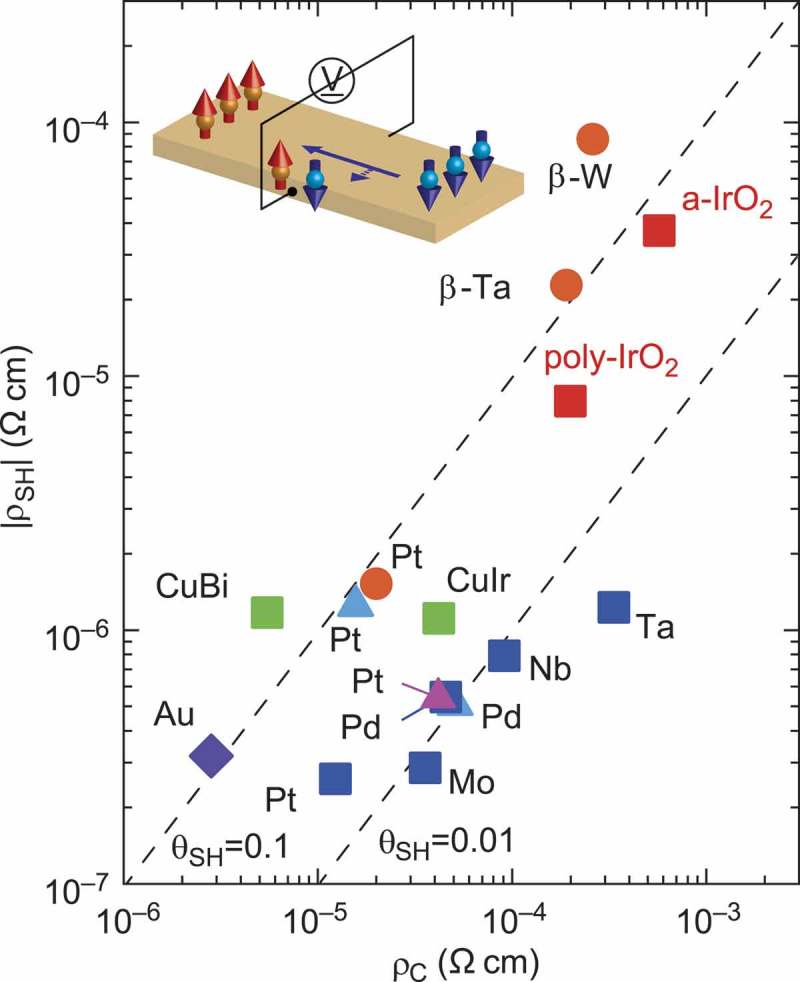
Experimentally measured values of spin Hall resistivity $\rho_{SH}$ for various metals are plotted as a function of electrical resistivity $\rho_{C}$. Replotted with permission from [61], © 2013 Macmillan Publishers Limited.

5*d* transition-metal oxides are recently suggested as alternative materials to realize large $\theta_{SH}$. The uniqueness of 5*d* oxides is characterized by the extremely strong SOC of 0.5–1 eV originating from the predominant 5*d* character of the conduction band [60]. In addition to the strong SOC, the localized character of *d* orbitals gives rise to a moderately high charge resistivity $\rho_{C}$ even in the metallic state. Typical $\rho_{C}$ values of conductive 5*d* Ir oxides such as IrO_2_ and SrIrO_3_ at room temperature are of the order of $10^{-4}$–$10^{-3}\;\Omega$cm, at least one order of magnitude higher than those of normal *s* metals. It is expected that a large spin Hall resistivity, which is roughly denoted as $\rho_{SH}≃\rho_{C}\theta_{SH}$ becomes larger in 5*d* oxides. Successful spin-current injection into a simple binary oxide, rutile IrO_2_, using a lateral spin-valve device geometry is reported [61]. ISHE with a remarkably high $\rho_{SH}≃$ 38 μΩcm as a metal not only in the polycrystalline state but also in the practically important amorphous state is demonstrated at room temperature. Figure 6 summarizes spin Hall resistivity $\rho_{SH}$ and electrical resistivity $\rho_{C}$ for a variety of metals with a large spin Hall angle $\theta_{SH}$ reported so far, together with that for IrO_2_. $\rho_{SH}$ of IrO_2_ is distinctly large compared with typical heavy metals and their alloys.

The large spin Hall resistivity can be applied to the spin Seebeck effect, which is denoted by

(2) $$E_{SSE}=g\rho_{C}\theta_{SH}\hat{s}\times j_{S},$$

where *g* is spin mixing conductance, spin-current transfer efficiency across interfaces [62] and $\hat{s}$ is a unit vector along the electron-spin polarization in the metallic layer; using Ir oxides as nonmagnetic layer is quite promising in spin Seebeck effect. However, Ir oxides have not been proved useful. The efficiency of Y_3_Fe_5_O_12_ (YIG)/IrO_2_ bilayers is almost one order of magnitude smaller than that of YIG/Pt [63]. While the reason of such poor performance is still an open question, one of the possibilities is the quality of the interfaces. Physics related to spin current and interfaces is attracting much attention and our understanding is improving day by day. After clarifying microscopic mechanism of the spin-current-induced thermoelectric conversion, there will be a chance to enhance it using 5*d*-electron oxides.

### 3.3. Role of interface with strong SOC: magnetic skyrmion

In Sec. 2.3, we have shown that to build an interface is one of the promising ways to enhance the figure of merit of the thermoelectric materials. We have just proposed in Sec. 3.2 that interfaces might play a key role in spin Seebeck effect as well. Recently, substantial progress has been made on thin films and heterostructures of 5*d* electron oxides [64–69]. In this context, we demonstrate spintronic function designed by fully utilizing the interface with strong SOC.

**Figure 7.**
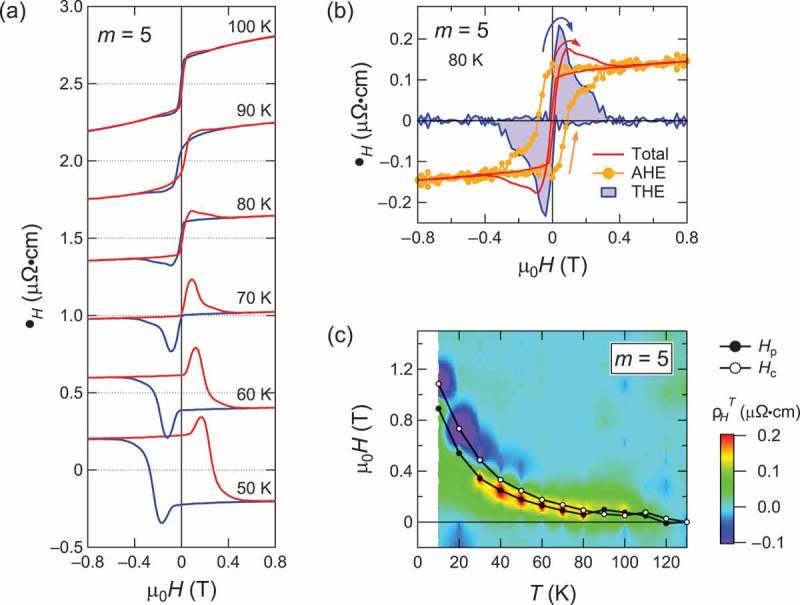
(a) Magnetic field dependence of Hall resistivity ($\rho_{H}$) of the (SrRuO_3_)_m_-(SrIrO_3_)_2_ bilayers (m=5) at various temperatures. (b) Contributions from AHE and THE of m=5 at 80 K. (c) Color map of topological Hall resistivity in the T−H plane for m=5. Black open and filled symbols represent coercive field ($H_{c}$) and the field at which topological Hall resistivity reaches its maximum ($H_{p}$), respectively. From [67], © 2016 American Association for the Advancement of Science.

Electron transport coupled with magnetism has attracted attention over the years. Among them, recently discovered is topological Hall effect (THE), originating from scalar spin chirality, that is, the solid angle subtended by the spins. THE is found to be a promising tool for probing the Dzyaloshinskii-Moriya (DM) interaction and consequent magnetic skyrmions [70]. This interaction arises from broken inversion symmetry and hence can be artificially introduced at interface; this concept is lately verified in metal multilayers. However, there are few attempts to investigate such DM interaction at interface through electron transport. How the transport properties couple with interface DM interaction is clarified by fabricating the epitaxial oxide interface [71]. Transport properties of bilayers consisting of *m* unit cells of ferromagnetic SrRuO_3_ and 2 unit cells of SrIrO_3_ are reported. An anomaly in the Hall resistivity in addition to AHE is observed as displayed in Figure 7(a); this is attributed to THE, which suggests existence of magnetic skyrmion. The clear anomaly in the Hall resistivity is observed only in the case of small *m* such as 4, 5, and 6. To precisely evaluate THE, AHE and THE are separated by measuring the Kerr rotation angle which is proportional to AHE. A representative data set of *m *= 5 at 80 K is plotted in Figure 7(b). One can fit the Hall resistivity by using the Kerr rotation angle to obtain AHE and can estimate THE by subtracting AHE from the total Hall resistivity. As a consequence, the topological Hall term as functions of both *T* and *H* is observed as shown in Figure 7(c). It is noted that the topological Hall term is observed in the wide range of the *T-H* plane, indicating the stability of the two-dimensional skyrmion. The topological term rapidly decreases with *m*, ending up with a complete disappearance at m=7. These results suggest that magnetic skyrmions of 10–20 nm are generated by DM interaction, which might be caused by both broken inversion symmetry at the interface and strong SOC of SrIrO_3_. The results established that the high-quality oxide interface enables us to tune the effective DM interaction; this can be a step toward future topological electronics.

While controlling AHE and THE with electric fields has been of interest as well, it has remained unachieved since an electric field tends to be screened in itinerant magnets. Electric-field modulation of both THE and AHE is also reported in oxide heterostructures consisting of ferromagnetic SrRuO_3_ and nonmagnetic SrIrO_3_ [72]. A clear electric field effect is observed only when SrIrO_3_ is inserted between SrRuO_3_ and a gate dielectric. The results establish that strong SOC of nonmagnetic materials such as SrIrO_3_ is essential in electrical tuning of these Hall effects and possibly other SOC-related phenomena.

We here point out that thermoelectric properties of topological spin textures including skyrmions start to attract attention. For instance, large anomalous Nernst effect is theoretically predicted in a skyrmion crystal [73]. Large magneto-thermopower in MnGe with topological spin texture is also experimentally observed [74]. The results shown in this section thus imply that Ir oxides can contribute to energy harvesting through their topological spin textures, while further studies are required on this new topic.

## 4. Summary

We reviewed recent advances in strongly correlated oxides as thermoelectric materials by focusing on two kinds of approaches. The first one is to enhance the figure of merit of ordinary thermoelectric properties by the following three ways: (i) to enhance the Seebeck coefficient by controlling the orbital degeneracy through carrier doping, (ii) to reduce the phonon thermal conductivity by the fluctuation of the orbital degree of freedom, and (iii) to reduce the phonon thermal conductivity by introducing the interface to the materials. The second one is to use new phenomena such as spin Seebeck effect and anomalous Nernst effect, both of which are dominated by SOC of materials. In Ir oxides containing 5*d* orbitals with strong SOC, we demonstrated the SOC-related phenomena which potentially contribute to energy harvesting: (i) correlated Dirac/Weyl semimetal, (ii) inverse spin Hall effect, and (iii) interface-driven magnetic skyrmions.

We would like to stress that the quest for the energy harvesting oxides is still on-going. While it is obviously important to increase the figure of merit itself, we sometimes do not know what is a proper figure of merit particularly in case of new thermoelectric phenomena [75]. For example, the figure of merit for spin Seebeck effect, $Z_{SSE}$, is defined in analogy with that of ordinary Seebeck effect. The actual performance, however, strongly depends on the device geometry as well as the interface quality, which are introduced in $Z_{SSE}$ in an implicit manner. In order to establish a proper performance index, we need basic understanding of materials with a practical application in mind; this is one of the important missions imposed on materials science community.
